# IGFBP-rP1, a potential molecule associated with colon cancer differentiation

**DOI:** 10.1186/1476-4598-9-281

**Published:** 2010-10-26

**Authors:** Wenjing Ruan, Shuzhen Zhu, Haibing Wang, Fangying Xu, Hong Deng, Yu Ma, Maode Lai

**Affiliations:** 1 Department of Pathology, School of Medicine, Zhejiang University, 388 Yuhangtang Road, Hangzhou 310058, Zhejiang Province, China

## Abstract

**Background:**

In our previous studies, we have demonstrated that insulin-like growth factor binding protein-related protein1 (IGFBP-rP1) played its potential tumor suppressor role in colon cancer cells through apoptosis and senescence induction. In this study, we will further uncover the role of IGFBP-rP1 in colon cancer differentiation and a possible mechanism by revealing responsible genes.

**Results:**

In normal colon epithelium, immunohistochemistry staining detected a gradient IGFBP-rP1 expression along the axis of the crypt. IGFBP-rP1 strongly expressed in the differentiated cells at the surface of the colon epithelium, while weakly expressed at the crypt base. In colon cancer tissues, the expression of IGFBP-rP1 correlated positively with the differentiation status. IGFBP-rP1 strongly expressed in low grade colorectal carcinoma and weakly expressed in high grade colorectal carcinoma. In vitro, transfection of PcDNA3.1(IGFBP-rP1) into RKO, SW620 and CW2 cells induced a more pronounced anterior-posterior polarity morphology, accompanied by upregulation with alkaline phosphatase (AKP) activity. Upregulation of carcino-embryonic antigen (CEA) was also observed in SW620 and CW2 transfectants. The addition of IGFBP-rP1 protein into the medium could mimic most but not all effects of IGFBP-rP1 cDNA transfection. Seventy-eight reproducibly differentially expressed genes were detected in PcDNA3.1(IGFBP-rP1)-RKO transfectants, using Affymetrix 133 plus 2.0 expression chip platform. Directed Acyclic Graph (DAG) of the enriched GO categories demonstrated that differential expression of the enzyme regulator activity genes together with cytoskeleton and actin binding genes were significant. IGFBP-rP1 could upreguate Transgelin (TAGLN), downregulate SRY (sex determining region Y)-box 9(campomelic dysplasia, autosomal sex-reversal) (SOX9), insulin receptor substrate 1(IRS1), cyclin-dependent kinase inhibitor 2B (p15, inhibits CDK4) (CDKN2B), amphiregulin(schwannoma-derived growth factor) (AREG) and immediate early response 5-like(IER5L) in RKO, SW620 and CW2 colon cancer cells, verified by Real time Reverse Transcription Polymerase Chain Reaction (rtRT-PCR). During sodium butyrate-induced Caco2 cell differentiation, IGFBP-rP1 was upregulated and the expression showed significant correlation with the AKP activity. The downregulation of IRS1 and SOX9 were also induced by sodium butyrate.

**Conclusion:**

IGFBP-rP1 was a potential key molecule associated with colon cancer differentiation. Downregulation of IRS1 and SOX9 may the possible key downstream genes involved in the process.

## Introduction

Insulin-like growth factor binding proteins (IGFBPs), described as essential modulators of IGF bioavailability, are a family of homologous proteins produced by many different tissues. IGFBPs have different molecular weight, amino acid composition, binding properties and distribution in biological fluids [1,2]. The classified IGFBPs, including IGFBP1-6, cooperate in regulating signals from insulin receptors and IGF receptors. The last few years have brought complexity, but also new vistas of insights into the IGFBPs superfamily with discovery of new IGFBPs(IGFBP7-15), who exhibit a low affinity for IGF. These IGFBPs were reclassified as IGFBP-related proteins (IGFBP-rPs), whose roles in intracellular signaling, cell growth and cell metabolism are emerging [3,4].

IGFBP-rP1 has been independently cloned in several cellular systems, and therefore has been previously identified as IGFBP7[4], meningioma associated cDNA 25 (mac25) [5,6], tumor-derived adhesion factor(TAF) [7], and prostacylin-stimulating factor(PSF) [8]. IGFBP-rP1 was cloned as a gene that downregulated in meningioma cell lines compared to primary cultures of benign leptomeningeal cells and as a senescence-associated gene from human mammary epithelial cells [5]. IGFBP-rP1 is particularly intriguing due to its implicated role in cancer. In vivo, different expression patterns of IGFBP-rP1 were found in various tumor types. Upregulated expression of IGFBP-rP1 was observed in acute lymphoblasma leukemia and in thyroid cancer[9,10]. Downregulated expression of IGFBP-rP1 was common in liver cancer, lung cancer and in meningiomas[11-13]. While both up- and downregulation of IGFBP-rP1 have been reported in breast and prostate cancer[14-17]. These findings make the role of IGFBP-rP1 complicated. In 1999, our laboratory purified the cDNA fragments of IGFBP-rP1 from colonic adenocarcinoma and normal mucosa cDNA subtraction libraries by suppressive subtractive hybridization (SSH) [18]. Our group presented evidence that methylation of exon 1 was the key regulatory mechanism silencing the expression of IGFBP-rP1 in colon cancer cell lines[19]. IGFBP-rP1 suppressed the proliferation, decreased the colony formation ability, and induced apoptosis and senescence in colorectal cancer cell lines [20,21]. The expression of IGFBP-rP1 was correlated with favourable prognosis in colon cancer patients[20]. All these findings strongly supported that IGFBP-rP1 played a potential tumor suppressor role against colorectal carcinogenesis. The tumor suppressive role of IGFBP-rP1 was also found in other types of cancer, including cervical cancer [22], osteosarcoma [22,23], prostate cancer [14,24], breast cancer [25], lung cancer[13], melanoma [26]and thyroid cancer[10].

The balance among proliferation, differentiation, senescence and apoptosis is tightly regulated to maintain homeostasis of colon epithelium. Neoplastic transformation arises from multiple defects in these processes. Malignant transformation is often characterized by both deregulated cell cycle, increased cell survival and loss of differentiation [27,28]. However, the role of IGFBP-rP1 in the differentiation in colon cancer cells remains elusive. The objectives of the present work were to uncover the role of IGFBP-rP1 in the differentiation of colon cancer and its possible responsible genes.

## Materials and methods

### Reagents

Dulbecco's Modified Eagle's Medium (DMEM) was purchased from GIBCO Laboratories (Grand Island, NY, USA). Fetal bovine serum (FBS) was purchased from HyClone Laboratories (Logan, UT, USA). Polyfect transfection reagent and RNeasy mini kit was purchased from QIAGEN (Hilden, Germany). G418 was purchased from Merck (Darmstadt, Germany). Trizol reagent was purchased from Invitrogen (Carlsbad, CA, USA). The polyclonal antibody of IGFBP-rP1 and of Actin were purchased from Santa Cruz Biologicals (CA, USA). The monoclonal antibody of IGFBP-rP1 and the recombinant IGFBP-rP1 protein were purchased from RD (Minneapolis, MN USA). The monoclonal antibody of carcinoembryonic antigen (CEA) and polyclonal antibody of caudal-related homeodomain transcription 2 (CDX2) were purchased from Cell Signaling (Danvers, MA, USA). Horseradish peroxidase-conjugated secondary antibodies were purchased from Zymed (San Francisco, CA, USA). Sodium Butyrate was purchased from Sigma Chemical Company (St. Louis, MO, USA). An alkaline phosphatase (AKP) kit was purchased from Pointe Scientific Inc.(Canton, MI, USA).

### Tissues

All 221 patients with colorectal carcinoma, 121 males and 100 females, were inhabitants of Xiaoshan District, Zhejiang Province, China. The age range of the patients was from 26 to 85 years (median, 59 years). All archival paraffin-embedded tissue blocks were collected from the Department of Pathology, Zhejiang University, and the People's No. 1 Hospital of Xiaoshan, and the Zhejiang Cancer Hospital from January 1990 to December 2000. Patient and treatment data were collected from patient records. The 221 patients had not received chemotherapy or radiotherapy prior to surgery. Paraffin-embedded tissue blocks were processed according to standard histologic procedures and stained with H&E. The type of histology: tubular adenocarcinoma (n = 164), papillary adenocarcinoma (n = 26), mucinous adenocarcinoma (n = 27), ring cell carcinoma, and undifferentiated carcinoma (n = 4), assessed by two experienced pathologists.

### Immunohistochemisty

The immunohistochemical staining was carried out as described[20]. Paraffin-embedded sections (5 μm thick) were first dewaxed in xylene and rehydrated with a graded ethanol series. Endogenous peroxidase was quenched by incubation in 3% H_2_O_2 _for 10 min at room temperature. Nonspecific binding was blocked by incubation in a 1:10 dilution of rabbit serum for 30 min at room temperature. Then, sections were incubated at 4°C overnight with a 1:200 dilution of goat polyclonal antibody against human IGFBP-rP1. After several washes in PBS, the sections were incubated with a 1:200 dilution of biotinylated rabbit IgG at room temperature for 30 min. Then, the slides were incubated in a 1:200 dilution of rabbit horseradish peroxidase at room temperature for 20 min. The peroxidase activity was visualized by incubating in 0.06% 3-3'-diaminobenzidine-H_2_O_2_. The sections were finally counter stained with hematoxylin. Immunohistochemical scores of each section were independently scored by two pathologists.

### Cell lines

Human colorectal carcinoma RKO, SW620, CW2 and Caco2 cell lines were maintained in DMEM supplemented with 10% FBS in a 37°C/5% CO_2 _atmosphere. PcDNA3.1(IGFBP-rP1)-RKO, PcDNA3.1(IGFBP-rP1)-SW620, PcDNA3.1(IGFBP-rP1)-CW2 transfectants and the empty vector transfectants were established as previously described [20,21], maintained in the same culture medium containing 200 μg/ml G418.

### Recombinant IGFBP-rP1 stimulation assay

RKO, SW620 and CW2 cells were seeded into 6-well plate at 2 × 10^5 ^cells/plate, 4 × 10^5 ^cells/plate, 4 × 10^5 ^cells/plate, respectively. After attachment for 24 hr, the cells were replaced by free culture medium with recombinant IGFBP-rP1 protein (For RKO cells, 10 μg/ml, For SW620 and CW2 cells, 4 μg/ml). Forty-eight hours after IGFBP-rP1 addition, cell morphology was observed and photographed. Expression of differentiation markers were performed on the cell lysates and supernatants.

### AKP activity assays

Cell lysates and the supernatants of the PcDNA3.1(IGFBP-rP1) transfectants, PcDNA3.1 transfectants, parental cells, and cells stimulated with recombinant IGFBP-rP1 protein were harvested as discussed above. AKP activity was determined according to the protocol of the kit. P-nitrophenyl phosphate disodium hexahydrate was used as a substrate. Synthetic alkaline phosphatase was used to construct a standard dilution curve.

### Western Blot

Antibodies directed against actin (1:5000), CEA (1:1000), CDX2 (1:1000) were used in Western blot analyses. Cell lysates (50 μg) were resolved in prepoured Tris-glycine SDS gels (Bio-Rad, Richmond CA), and transferred to a nitrocellulose membrane (Bio-Rad). Blots were blocked in 5% nonfat milk in TBST (TBS buffer containing 0.1% tween), incubated with the primary antibody overnight at 4°C, washed in TBST, and then incubated with appropriate secondary antibody for 1 hr at room temperature. Antibody binding was detected using enhanced chemiluminescence reagent according to the manufacturer's instructions.

### RNA isolation and microarray hybridization

We applied Affymetrix HG133 plus 2.0 chip to detect differentially expressed genes transfected by PcDNA3.1(IGFBP-rP1) in RKO cells. As technical replication of Affymetrix chip were more than 99% consistent, we performed biological replications to reduce sampling variability. We selected three single-cell clones of PcDNA3.1(IGFBP-rP1) transfectants, identified as RP5, RP6, RP7, also three control vector transfectants as control, named as EV5, EV6, EV7. Cells were divided into three paired groups, RP5 VS EV5, RP6 VS EV6, RP7 VS EV7. The paired cells were cultured in the same condition and harvested at the same time to minimize gene expression changes due to cell culture conditions. Total RNA was isolated from cells using RNeasy mini kit (Qiagen, Germany). RNA was quantitated by UV absorbance at 260 and 280 nm and assessed qualitatively using an RNA LabChip and Bioanalyzer 2100 (Agilent, Palo Alto, CA). To generate a biotinylated probe, cDNA was synthesized (Superscript cDNA synthesis kit, Invitrogen, San Diego, CA) from 4 μg RNA using an oligo(dT) primer with a T7 RNA polymerase promoter at the 5' end. The cDNA was made double stranded and used in an in vitro transcription reaction (Enzo Diagnostics) with T7 RNA polymerase to synthesize biotinylated product for hybridization to Affymetrix GeneChips HU133 plus 2.0 using the Affymetrix recommended protocolhttp://www.affymetrix.com/support/technical/manual/expression_manual.affx. Human HG-U133 plus 2.0 chips (Affymetrix, Inc.) were hybridized with 15 μg of fragmented labeled cRNA overnight at 45°C, washed (Genechip Fluidics Station 400; Affymetrix), and scanned (GeneArray Scanner; Affymetrix) according to Affymetrix protocols. Together 6 chips were performed.

### Microarray data analysis

Scanned images of microarray chips were analysed by the GeneChip Operating Software (GCOS1.2) from Affymetrix. The total HG-U133 Plus 2 signal was normalized to an arbitrary signal intensity value of 500. Differentially expressed genes between IGFBP-rP1 transfectants and the controls in the groups were identified using the GCOS change algorithm and Rank Products (RP) following RMA (Robust Multiarray Analysis, One-sided Wilcoxon's Signed Rank Test). A total of three possible pairwise comparisons were conducted (RP5 VS EV5, RP6 VS EV6, RP7 VS EV7). For each comparison between the groups, the number of increase and decrease calls of each probe set was calculated using MS Excel and probe sets with the highest number of consistent changes among all samples were identified. Using these criteria, 115 genes showed statistically significant alterations in expression in at least two replicate studies. Gene lists were uploaded to GOTM, and functional annotation was performed. Further information on genes were obtained from public databases, such as NCBI. Hierarchical clustering was done using the clustering function (condition tree) in GeneSpring7.2 (Silicon Genetics, Inc., Redwood City, CA).

### Real-time RT-PCR(rtRT-PCR)

Three micrograms of total RNA was reverse transcribed by M-MuLV reverse transcriptase with Oligo(dT)16 as a primer. RtRT-PCR was performed on the ABI PRISM 7500 sequence detection system (Applied Biosystems, Foster City, CA, USA) by monitoring the increase of fluorescence by the binding of SYBR Green (Applied Biosystems) to double-stranded DNA. Dissociation analysis was performed at the end of each PCR reaction to ensure there was only specific product. Settings for the PCR thermal profile were: initial denaturation at 95°C for 1 min, followed by 40 amplification cycles of 95°C for 15 sec, annealing at 59°C for 20 sec, and elongation at 72°C for 40 sec. The primer sequences used are listed in Table 1. Each PCR was run in triplicate. For quantification of gene expression changes, ^the ΔΔ^Ct method was used to calculate relative -fold changes normalized against the GAPDH gene.

**Table 1:** Primer sequences used in rtRT-PCR

Gene	Primer	Product (bp)	Design
AREG	5' CGGGAGCCGACTATGACTACTC 3' 5' GGGCTTAACTACCTGTTCAACTCTG 3'	100	[29]
IRS1	5'CAGAGGACCGTCAGTAGCTCAA 3' 5'GGAAGATATGAGGTCCTAGTTGTGAAT3'	135	[29]
CDKN2B	5'CACCGTTGGCCGTAAACTTAAC 3' 5'TAATGAAGCTGAGCCCAGGTCT 3'	96	[30]
ID1	5' ATTTCTTCTCGTTTTCACAGGC 3' 5' TCGGTCTTGTTCTCCCTCAG 3'	174	[31]
SOX-9	5'AGGTGCTCAAAGGCTACGACT 3' 5'AGATGTGCGTCTGCTCCGTG3'	359	[32]
STC1	5' AACCCTGAAGCCATCACTG 3' 5' GCTTCGGACAAGTCTGTTATAG 3'	78	[33]
TACSTD1	5'TGTTTGGTGATGAAGGCAGA 3' 5' ACGCGTTGTGATCTCCTTCT 3'	324	Primer 5.0
TAGLN	5'ATCCTGTCTGTCCGAACCC 3' 5' GCACTATGATCCACTCCACC 3'	184	Primer 5.0
FRMD4A	5' TGGCTTCTCACTTCAATCT 3' 5' CCACGGGTCCTGACTTTT 3'	134	Primer 5.0
IER5L	5' AGCCCTTGGAGCCTCTGCA 3' 5' CGGAGCCAAAGATGGAGATCA 3'	214	Primer 5.0
SP140	5' TGATCCTCCAAGAATACG 3' 5' ACAAGTGTCGCAACAGAA 3'	141	Primer 5.0
SYN1	5'GTGTCAGGGAACTGGAAGACC 3' 5'TGAGCGGCATGGAGGAAC3'	192	Primer 5.0
LAMB1	5' GAAGACGGGAAGAAAGGG 3' 5' GTCGAGGTCACCGAAAGC 3'	245	Primer 5.0
KERATIN 7	5' CAATGAGACGGAGTTGACAG 3' 5' ACGCTGGTTCTTGATGTT 3'	304	Primer 5.0
KERATIN 8	5' TTGCAGATGCCGAGCAGCGT 3' 5' TGGGCTGAGGGCTAGGGCTG 3'	576	Primer 5.0
PROFILIN2	5' ATTGTCGGCTACTGCG 3' 5' ATTGTATGTTGGCTCC 3'	237	Primer 5.0
GAPDH	5' ACGGATTTGGTCGTATTGGG 3' 5' CGCTCCTGGAAGATGGTGAT 3	213	Primer 5.0

### Cell differentiation induction assays

For sodium butyrate treatments, Caco2 cells were seeded into 60-mm culture dishes at 3 × 10^5 ^cells/plate. After 24 hr, cells were cultured in medium contained 4 mmol/L sodium butyrate. The cells were harvested at the time points of 24 hr, 48 hr, 72 hr after sodium butyrate stimulation. Expression of differentiation markers and IGFBP-rP1 were performed in the cell lysates and supernatants.

### ELISA

The ELISA method was carried out as described [34]. Briefly, wells of microtiter plates were coated (for 18 hr at 4°C) with 100 ng/ml IGFBP-rP1 polyclonal antibody in 100 μl of coating buffer (0.05 M Na_2_CO_3 _and 0.05 M NaHCO_3_, pH 9.6) and were then blocked with 2% BSA in PBS for 1 hr at 37°C. Samples were diluted with 0.5% BSA (1:1) and a total of 100 μl was loaded in duplicates and incubated for 2 hr at room temperature, followed by the addition of 100 μl IGFBP-rP1 monoclonal antibody (200 ng/ml) for an additional 2 hr at room temperature. HRP-conjugated goat anti- mouse IgG (1:20,000) in blocking buffer was added (for 1 hr at room temperature) and the reaction was visualized by the addition of 100 μl of the chromogenic substrate (3,30,5,50- tetramethylbenzidine) for 30 min. The reaction was stopped with 100 μl H_2_SO_4 _and absorbance at 450 nm was measured with a reduction at 630 nm using ELISA plate reader. Plates were washed five times with washing buffer (PBS, pH 7.4, containing 0.1% (v/v) Tween 20) after each step. As a reference for quantification, a standard curve was established by a serial dilution of recombinant IGFBP-rP1 protein, ranging from 150 ng/ml to 1 ng/ml.

### Statistical analysis

Statistical package SPSS (version 11.0) was applied. The Chi-square test was used to analyze the ranked data in tissue samples. Student't test was used in the cell line experiments. A value of P < 0.05 was considered statistically significant.

## Results

### Expression pattern of IGFBP-rP1 in normal colonic epithelium and colorectal carcinoma

**Figure 1:**
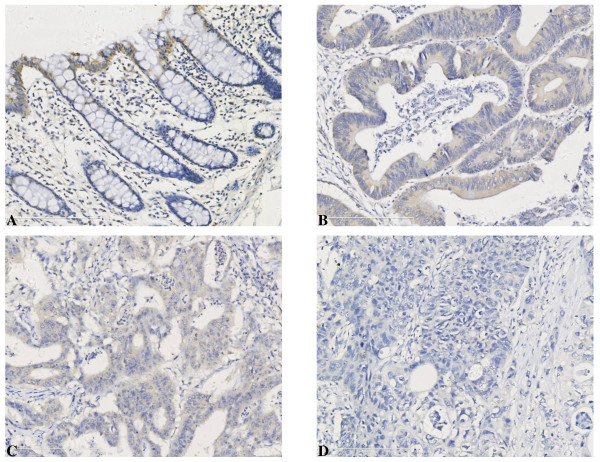
**IGFBP-rP1's expression in normal colonic epithelium and primary colon cancer. **Representative images of IGFBP-rP1 detection by immunohistochemistry staining in human colonic tissue sections. Magnification: _200. A. IGFBP-rP1 expression localized to the terminally differentiated epithelium at the luminal surface of the bowel and at the tops of the colonic crypts. B. Strong expression of IGFBP-rP1 in low grade colorectal adenocarcinoma. C. Media expression of IGFBP-rP1 in medium grade colorectael adenocarcinoma. D. Weak expression of IGFBP-rP1 in high grade colorectal adenocarcionma.

The expression pattern of IGFBP-rP1 in the human colonic epithelium was analyzed using immunohistochemistry staining. The intensity of IGFBP-rP1 staining varied from the basal compartment to the surface epithelium. Epithelial cells at the surface displayed a very strong IGFBP-rP1 expression, whereas IGFBP-rP1 staining was much weaker at the crypt base (Figure 1A). These results indicated that IGFBP-rP1 expression is stronger in the proliferating and differentiating compartment of colonic epithelium. Interestingly, IGFBP-rP1 expression is also well maintained in colorectal cancer. Comparison of the IGFBP-rP1 expression level performed in paired cancerous and normal tissues indicated that IGFBP-rP1 was overexpressed in colon cancer tissue compared to paired normal tissue (P = 0.001, Table 2). When analysed the expression pattern of IGFBP-rP1 in cancer we found that IGFBP-rP1 was strongly expressed in the well differentiated colorectal adenocarcinoma, while weakly expressed in poorly differentiated colorectal adenocarcionma (Figure 1B-D). We then analyzed the correlation between the expression of IGFBP-rP1 and the differentiation status of the colorectal carcinoma. The staining of IGFBP-rP1 was divided into four grades (0-2), representing lack and mild of staining (0), intermediate staining (1), and strong staining (2). The histologic grade of colorectal carcinoma was divided into two grades: low grade (gland formation >50% in tubular adenocarcinoma and papillary carcinoma) and high grade (gland formation <50% in tubular adenocarcinoma, mucinous adenocarcinoma, signet ring cell carcinoma, and undifferentiated carcinoma). As a result, the higher expression of IGFBP-rP1 was closely related with good differentiation status (Table 3). The difference was found to be statistically significant (P = 0.006).

**Table 2:** Comparison of IGFBP-rP1's expression in paired cancerous and normal tissues

IGFBP-rP1 expression in Cancerous tissue	IGFBP-rP1 expression in paired Normal tissue			Total	Significance
			
	0	1	2		
0	1	4	3	8	
1	26	87	21	134	
2	5	26	24	55	
Total	32	117	48	197	0.001*

The staining of IGFBP-rP1 was divided into four grades (0-2), representing lack and mild of staining (0), intermediate staining (1), and strong staining (2). *The P value was calculated by McNemar-Bowker Test.

**Table 3:** Correlation between IGFBP-rP1 expression and differentiation in colorectal carcinoma

IGFBP-rP1 expression	Grade^a^		Total	Significance
			
	Low	High		
0	3	9	12	
1	102	46	148	
2	43	18	61	
Total	148	73	221	0.006*

The staining of IGFBP-rP1 was divided into three grades (0-2), representing lack and mild of staining (0), intermediate staining (1), and strong staining (2).

^a^histologic grade: low grade (gland formation >50% in tubular adenocarcinoma and papillary carcinoma) and high grade (gland formation <50% in tubular adenocarcinoma, mucinous adenocarcinoma, signet ring cell carcinoma, and undifferentiated carcinoma).*The P value was calculated by chi-square test.

### Morphology change of colorectal cancer cells induced by IGFBP-rP1

**Figure 2:**
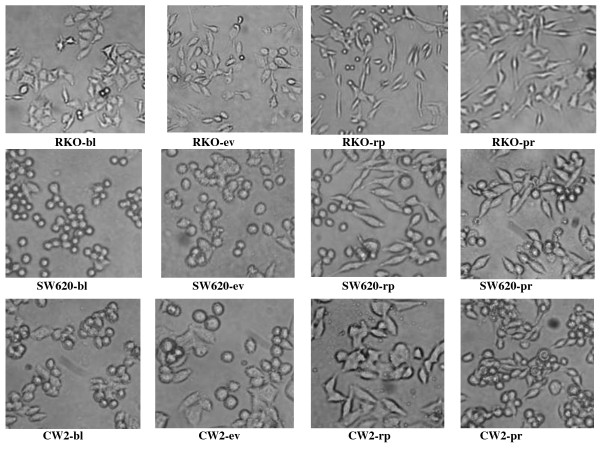
**Morphology of IGFBP-rP1 expressed cells and the control cells **. Three colon cancer cell lines (RKO, SW620, CW2) were cultured in DMEM medium. Phase-contrast micrographs of various cells were photographed. A, Parental blank cells (-bl). B, Cells transfected with PcDNA3.1 empty vector (-ev). C, Cells transfected with PcDNA3.1(IGFBP-rP1) (-rp). D, Cells treated with recombinant IGFBP-rP1 protein for 48 hr (-pr, 4 μg/ml for SW620 and CW2, 10 μg/ml for RKO,). RKO cells, Magnification: 100. SW620, CW2 cells, Magnification: 200.

Under light microscopy, we found that control vector transfected RKO cells exhibited the same polygon-like morphology as the parental RKO cells, while PcDNA3.1(IGFBP-rP1)-RKO transfectants exhibited elongated morphology with a more pronounced anterior-posterior polarity. As in SW620 and CW2 cells, we found that the parental and the control vector transfected cells exhibited the round cell morphology, while PcDNA3.1(IGFBP-rP1) transfectants were flatter and spindle shaped. Similar morphological changes were seen when recombinant IGFBP-rP1 protein was added into the culture medium of the parental cells (Figure 2).

### Regulation of well known differentiation markers by IGFBP-rP1

**Figure 3:**
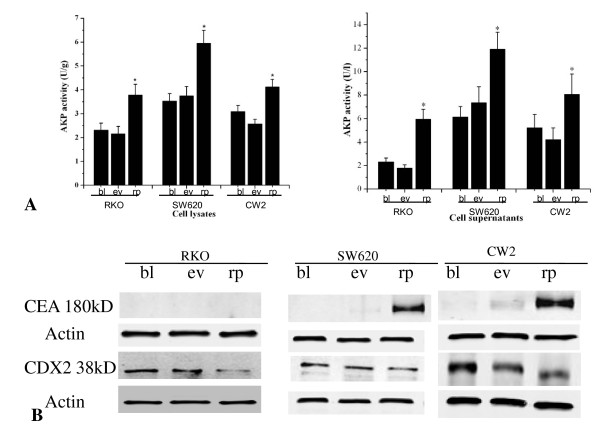
**Well known differentiation marker analysis after IGFBP-rP1 transfection. **The experiments were conducted in three colon cancer cell lines: RKO, SW620, CW2. The parental blank cells (-bl), PcDNA3.1 empty vector transfectants (-ev), and PcDNA3.1(IGFBP-rP1) transfectants (-rp), were cultured in DMEM medium. A:Total cell lysates and supernatants were harvested and assayed for alkaline phosphatase activity assessing. Results represent the mean (± SD) of three experiments.*p < 0.05. B. Total cell lysates were harvested and assayed for CEA, CDX2 expression. Also shown are the corresponding reprobings of these blots with actin as a loading control. This blot is representative of those obtained when the experiment was performed six times.

**Figure 4:**
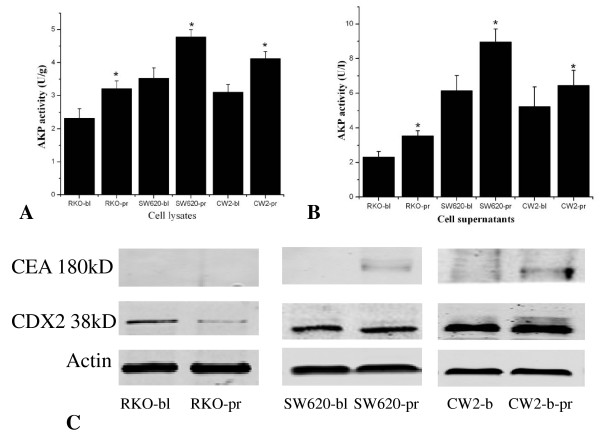
**Well known differentiation marker analysis after recombinant IGFBP-rP1 stimulation**. The experiments were conducted in three colon cancer cell lines: RKO, SW620, CW2. After attachment for 24 hr, the cells were then stimulated with (-pr, for RKO cells, 10 μg/ml, For SW620 and CW2 cells, 4 μg/ml) or without recombinant IGFBP-rP1 protein (-bl) in medium without FBS. Forty-eight hours after IGFBP-rP1 addition, expression of differentiation markers were performed in the cell lysates and supernatants. A:Total cell lysates and supernatants were harvested and assayed for AKP activity assessing. Results represent the mean (± SD) of three experiments.*p < 0.05. B. Total cell lysates were harvested and assayed for CEA, CDX2 expression. Also shown are the corresponding reprobings of these blots with actin as a loading control. This blot is representative of those obtained when the experiment was performed six times.

To further explore the role of IGFBP-rP1 in colon cancer differentiation, the expression level of several colonic epithelial cell differentiation markers, including AKP activity, CEA and CDX2, were determined. The experiments were done in three colon cancer cell lines, RKO, SW620 and CW2 cells. The PcDNA3.1(IGFBP-rP1) transfectants showed increased AKP activity compared with the empty vector transfectants and the parental cells, in both the cell lysates and the supernatants of the cells (Figure 3A). The differences were statistically significant with p values < 0.05. PcDNA3.1(IGFBP-rP1)-SW620 and PcDNA3.1(IGFBP-rP1)-CW2 transfectants showed increased CEA expression, but no obvious regulation on CDX2. As to RKO cells, no detectable regulation of CEA but a little downregulation of CDX2 was observed after transfection with IGFBP-rP1 cDNA (Figure 3B). The addition of recombinant IGFBP-rP1 to the superculture medium of the cells could mimic the effects of the transfection of IGFBP-rP1, although the extent was not that high (Figure 4).

### Gene expression profiles

**Figure 5:**
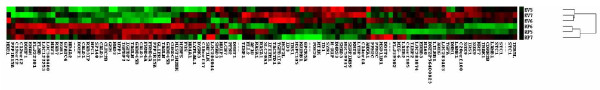
**Hierarchical clustering from six samples, based on the differentially expressed 115 probesets. **RNA samples from three independent biological replicate studies of RKO cells transfected with either PcDNA3.1(IGFBP-rP1) (three single cell clones, named RP5, RP6, RP7)or with either empty vector control (three single cell clones, named as EV5, EV6, EV7) were labeled and hybridized to Affymetrix U133 plus 2.0 GeneChips. Gene expression data were analyzed with GCOS1.2. One hundred and fifteen differentially expressed probesets according to a signal intensity cutoff protocol described in the text were selected for hierarchical clustering using GeneSpring 7.2. Genes with similar expression profiles were grouped together and the resulting gene tree is shown. Strong replicate clustering was shown. The left three lines mean the results in EV 5, EV6, EV7. The right three lines mean the results inRP5, RP6, RP7. Red represents up-regulation, green represents down-regulation. Black indicates no change. Quantitative data for the magnitude of each gene expression change, together with gene descriptions are shown in Table 4.

**Figure 6:**
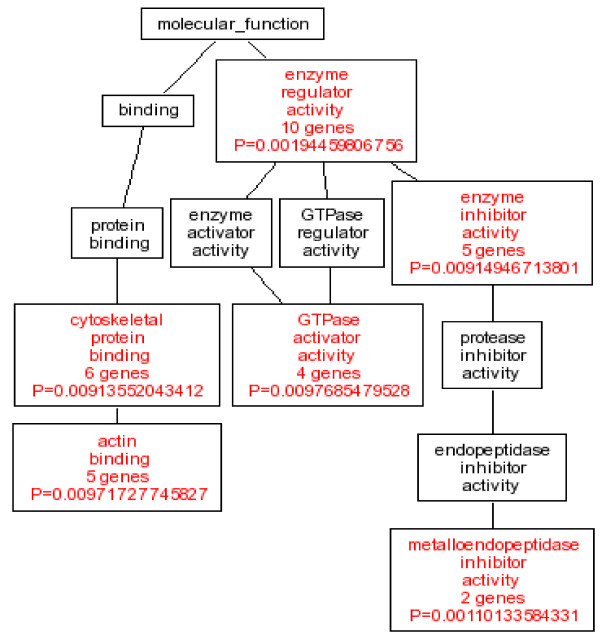
**Directed Acyclic Graph (DAG) of the enriched GO categories**. The 115 gene probe sets differentially induced by IGFBP-rP1 were put to the gene ontology analysis. Shown is the result of DAG analysis, a directed graph with no directed cycles. The graph showed the significance of differently expressed genes and more specifically, the gene ontology terms present in numbers that are above random chance. P < 0.01 and at least 2 genes (which are colored red) and their non-enriched parents (which are colored black).

Microarray analysis was performed to analyze the gene expression induced by IGFBP-rP1 in three PcDNA3.1(IGFBP-rP1) clones and three control clones. The reproducible 115 gene expression levels were used as a gene list and experiments were organized by both individual samples (to test sample reproducibility) as well as groups (IGFBP-rP1 transfectants vs control) (Figure 5). Collectively, seventy-eight genes reproducible in two clones were identified. Based on the gene ontology classification and the information in PUBMED, we classified the genes into 6 categories, including cytoskeleton and actin binding, extracellular, cell proliferation and differentiation, enzyme regulation, nuclear transcription, and others. Quantitative data for the magnitude of each gene expression change, together with gene descriptions are shown in Table 4. We utilized the Directed Acyclic Graph (DAG) of the enriched GO categories (Figure 6), which demonstrated that enzyme regulator activity genes, cytoskeletal proteins, and actin binding genes showed the highest differential expression and significance ((P < 0.01). Among them sixteen genes were reproducible in 3 clones, including IGFBP-rP1, Transgelin (TAGLN), SP140 nuclear body protein (SP140), FERM domain containing 4A (FRMD4A), caldesmon 1 (CALD1), neuron navigator 3 (NAV3), SRY (sex determining region Y)-box 9(campomelic dysplasia, autosomal sex-reversal) (SOX9), stanniocalcin 1 (STC1), amphiregulin(schwannoma-derived growth factor) (AREG), inhibitor of DNA binding 1, dominant negative helix-loop-helix protein (ID1), insulin receptor substrate 1 (IRS1), tumor-associated calcium signal transducer 1 (TACSTD), immediate early response 5-like (IER5L), cyclin-dependent kinase inhibitor 2B (p15, inhibits CDK4) (CDKN2B), synapsin I (SYN1) and laminin, beta 1 (LAMB1).

**Table 4:** Seventy-eight reproducible differentially expressed genes induced by IGFBP-rP1 in RKO cells

the cytoskeleton and actin binding	Nuclear transcription
[NM_004342] CALD1 (1, 1.4, 0.7) *	[NM_024496] C14orf4 (-0.3,0.1(NC),-0.3)
[NM_001457] FLNB (0.4,1.4,-0.3)	[NM_032883] C20orf100 (-0.8,-0.2(NC),-0.6)
[NM_018027] FRMD4A (0.5,0.6,0.5) *	[NM_001964] EGR1 (0.7,0.4,-0.1)
[NM_005556] KRT7 (-0.2,-0.3,-0.2 (NC**))	[NM_012081] ELL2,(0.2,-0.6,-1.1)
[NM_002273] KRT8(-0.7,-0.3 (NC),-1)	[NM_005252] FOS (1.1,0.6,-0.5)
[NM_002281] KRTHB1 (-3.8,0.7,-1.3)	[NM_017445] H2BFS (0.5,0.7,0)
[NM_002628] PFN2 (0.3,-0.7,-0.8)	[NM_080593] HIST1H2BK (0.5,0.6,-0.1(NC))
[NM_006950] SYN1 (-1.7,-1.5,-2.7) *	[NM_002165] ID1 (-1.2,-1.2,-1) *
[NM_001001522] TAGLN (1,1.6,0.6) *	[NM_002167] ID3 (-0.3(NC),-0.9,-0.7)
**Extracellular related**	[NM_014903] NAV3 (0.9,0.4,0.6) *
[NM_001657] AREG (-2.7,-1.6,-0.4) *	[NM_002135] NR4A1 (0.6,0.9,-0.6 (NC))
[NM_004406] DMBT1 (0.5,-1.4,-1)	[NM_006186] NR4A2 (0.4,1,-0.4)
[NM_006851] GLIPR1 (0.3,0.1,0)	[NM_003489] NRIP1 (-0.9,0.4, -0.3)
[NM_001553] IGFBP-rP1 (6.6,5.6,5.9) *	[NM_005902] SMAD3 (-0.5,0.1,-0.6)
[NM_003155] STC1 (-2.7,-3.8,-1.5) *	[NM_001005176] SP140 (1.1,1.3,0.6) *
[NM_003236] TGFA (-0.3, -0.5, -0.5)	[NM_006022] TSC22D1 (-0.3(NC), -0.6,-0.5)
[NM_003254] TIMP1 (-0.1(NC),-0.3, -0.6)	[NM_024836]ZNF672 (0.5,0.9,-0.1(NC))
[NM_003255] TIMP2 (-4.5,-0.3 (NC),-1)	**Others**
[NM_022164] TINAGL1 (-0.9,0.9,-0.4)	[NM_032744] C6orf105 (-5 (NC),-1.5,-2.3)
**Cell proliferation and differentiation**	[Hs.569345]C14orf34 (1.3,1.6,0.1(NC))
[NM_000700] ANNEXIN A1 (-1.8,-1.2.-0.5(NC))	[NM_005127] CLEC2B (0.9,1.3,0.4(NC))
[NM_004936] CDKN2B (-0.8,-1.7, -0.8) *	[NM_001004023] DYRK3 (0.8,0.9,-0.3(NC))
[NM_018482] DDEF1 (0.3,0.6,0.1(NC))	[NM_001008493] ENAH (0.4,0.4,0.1(NC))
[NM_015675] GADD45B (1.1,0.8,0.2 (NC))	[NM_001005915] ERBB3 (-0.8,0.2(NC),-0.4)
[NM_003641] IFITM1(-0.7,-1.5,-0.4 (NC))	[NM_005330] HBE1 (4.3,-4.6,-1.5)
[NM_001025242] IRAK1 (-0.6 (NC), -1.5,-0.6)	[NM_005525] HSD11B1 (-3.1,-1.1,1.7)
[NM_014330] PPP1R15A (0.7,0.5, -0.2)	[NM_203434] IER5L (-0.7,-0.7,-0.7) *
[NM_173354] SNF1LK (-0.5,-0.2,0.5)	[NM_005544] IRS1 (-1.4,-0.7,-0.9) *
[NM_000346] SOX9 (-1.9,-1.5,-1.1) *	[NM_002288] LAIR2 (-0.9,-1.3,0.4)
[NM_002354] TACSTD1 (-0.7,-1.4,-1.9) *	[NM_002291] LAMB1 (-1.1,-0.6,-1) *
**Enzyme regulated**	[NM_006669] LILRB1(-1.5,-1.2,-0.1 (NC))
[NM_004753] DHRS3 (-2.4,0.1 (NC),-1.6)	[NM_021070] LTBP3 (-1.1,0.5(NC),-0.6)
[NM_001005336] DNM1 (-0.6,-0.4,-0.3(NC))	[NM_138794] LYPLAL1 (0.4,0.4,-0.3(NC))
[NM_005261] GEM (0.8,0.5,0.1 (NC))	[NM_024979] MCF2L (-0.1(NC),-2.6,-3.8)
[NM_015590] GPATCH4 (0.3,0.4,-0.1(NC))	[NM_006818] MLLT11 (1,-1.4,-1)
[NM_003979] GPRC5A (-1.2,-0.1,-0.7)	[NM_176870] MT1K (-0.5,-0.2(NC),-0.6)
[NM_004637] RAB7 (0.5,0.5,-0.2)	[NM_000271] NPC1 (0.6,0.8,0.1)
[NM_002890] RASA1 (0.3,-0.4,-0.5)	[NM_002599] PDE2A (-2(NC),-1.7,-2.4)
[NM_007211] RASSF8 (0.4(MI***),1.9,2.5)	[NM_018444] PPM2C (-0.6,-0.5,0.2(NC))
[NM_005168] RND3 (1,0.7, 0.1(NC))	[NM_004155] SERPINB9 (-0.6,0.3 (NC),-0.9)
	[NM_005415] SLC20A1 (0.2(NC), -0.3,-0.5)
	[NM_003364] UPP1(0.4,1,0.1(NC))

Numbers in parentheses are signal log ratio(log_2_fold change) in three clones, respectively. Positive number indicates up-regulation in IGFBP-rP1-RKO transfectants vs empty vector-RKO transfectants, whereas negative number indicates down-regulation in IGFBP-rP1-RKO transfectants vs empty vector-RKO transfectants. *genes reproducible in 3 clones; **NC: no change or great interruption; ***MI: Marginal Increase.

### Validation of a Subset of the differentially expressed genes by RT-PCR

**Figure 7:**
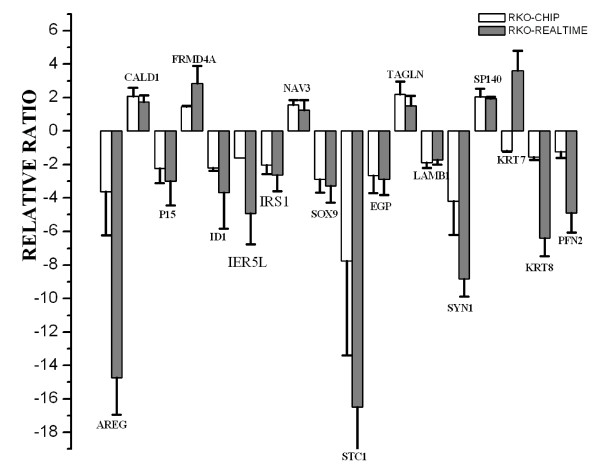
**Verification of selected microarray results by rtRT-PCR**. The 16 reproducible differentially-expressed genes in 3 clones induced by IGFBP-rP1 in RKO cells and the differentially-expressed cytoskeleton associated genes keratin 7, keratin 8, profilin2 analysed by chip were selected for rtRT-PCR validation. RtRT-PCR was performed on RNA extracted from IGFBP-rP1-RKO transfectants and the control cells. Delta-delta Ct method was used to calculate relative fold changes normalized against the GAPDH gene. The relative fold change for IGFBP-rP1 from rtRT-PCR is 2.1 × 10^6^. Results represent the mean (± SD) of six experiments.

**Figure 8:**
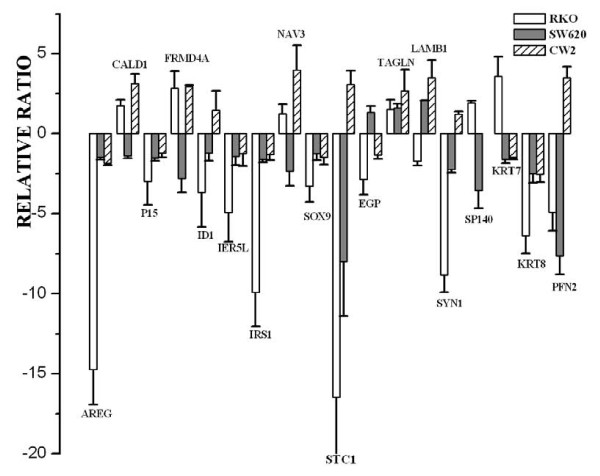
**Verification of the differentially expressed genes in SW620-IGFBP-rP1 transfectants and CW2-IGFBP-rP1 transfectants**. The expression of the 16 reproducible genes in 3 clones and the differentially-expressed cytoskeleton associated genes keratin 7, keratin 8, profilin2 analysed by chip were also analysed by rtRT-PCR in IGFBP-rP1-SW620 and IGFBP-rP1-CW2 transfectants versus empty vector control cells. RtRT-PCR was performed on RNA extracted from the transfectants and the control cells. Delta-delta Ct method was used to calculate relative fold changes normalized against the GAPDH gene. Results represent the mean (± SD) of six experiments.

To examine the reliability of the microarray data, the 16 reproducible genes in three clones, together with the cytoskeletal protein binding genes and actin binding genes keratin 7 (KRT7), keratin 8 (KRT8) and profilin2 (PFN2) were chosen for additional validation, by rtRT-PCR(Figure 7). The Affymetrix chip data and the rtRT-PCR data showed a good correlation. The relative fold change for IGFBP-rP1 measured by rtRT-PCR is 2.1 × 10^6 ^and 97 as measured by microarray chips. Eleven transcripts examined (CALD, P15, FRMD4A, ID1, IRS1, NAV3, SOX9, EGP, TAGLN, LAMB1, SP140) demonstrated concordant levels of differential expression between GeneChip and RT-PCR analysis. Six additional genes (AREG, KRT8, IER5L, PFN2, STC1, SYN1) showed qualitatively concordant, but quantitatively different expression fold changes between gene chip and RT-PCR analysis. One gene (KRT7) showed qualitatively different expression fold changes. These difference are more likely explained by the use of the ^ΔΔ^CT method to calculate relative expression levels in the RT-PCR assay. To explore whether these differentially expressed genes could also be induced by IGFBP-rP1 in other colon cancer cells, we analyzed the expression change of these genes in PcDNA3.1(IGFBP-rP1)-SW620 and PcDNA3.1(IGFBP-rP1)-CW2 transfectants. We found that IGFBP-rP1 could upreguate TAGLN, downregulate SOX9, IRS1, P15, AREG, IER5L, KRT8 in these colon cancer cells(Figure 8), indicating that these genes may be the important IGFBP-rP1 responsible genes in colon cancer.

### Gene expression changes during differentiation induced by butyrate in Caco2 cells

**Figure 9:**
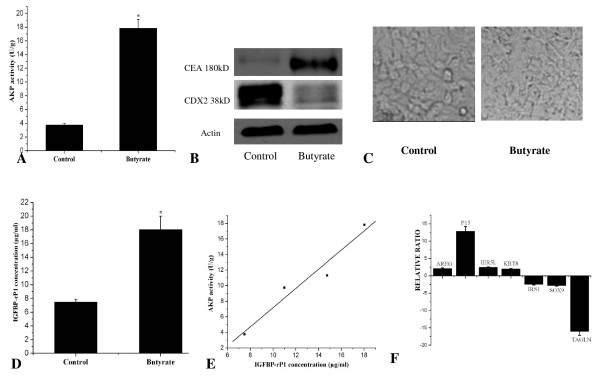
**Gene expression changes during differentiation induced by butyrate in Caco2 cells**. Caco2 cells were seeded into 60-mm culture dishes at 3 × 10^5 ^cells/plate. After 24 hr, cells were cultured in 4 mmol/L sodium butyrate for additional 24 hr, 48 hr, 72 hr. Results are averages of triplicate determinations. A. Total cell lysates with (72 hr point) or without butyrate stimulation were harvested and assayed for AKP activity assessing. Results represent the mean (± SD) of three experiments.*,**p < 0.05. B. Total cell lysates with (72 hr point) or without butyrate stimulation were harvested and assayed for CEA, CDX2 expression Also shown are the corresponding reprobings of these blots with actin as a loading control. This blot is representative of those obtained when the experiment was performed six times. C. Phase-contrast micrographs of cells with (72 hr point) or without butyrate stimulation were photographed. D. IGFBP-rP1 level analyzed by ELISA assay with (72 hr point)or without butyrate stimulation *P < 0.05. E. The relationship between AKP activity and the IGFBP-rP1 level during the sodium butyrate stimulation process. The correlation coefficient (r) = 0.945, P = 0.028. F. rtRT-PCR analysis of the IGFBP-rP1 induced differentially expressed genes (AREG, KRT8, P15, IER5L, IRS1, SOX9, TAGLN) in Caco2 cells with or without butyrate stimulation.

The sodium butyrate induced Caco2 cell differentiation model has been widely used. The phenomenon was also verified in our study. After 72 hr stimulation, the significant upregulation of AKP activity and CEA expression confirmed that differentiation occurred (Figure 9A, B). Loss of cell membrane integrity and loss of cell attachment were observed at the 72 hr stimulation point (Figure 9C), although there is no detectable morphology change at the 24 hr and 48 hr stimulation point. We want to use this model to study the IGFBP-rP1 regulation in this differentiation process. We found that IGFBP-rP1 protein increased during sodium butyrate-induced terminal cell differentiation (Figure 9D). The IGFBP-rP1 expression level showed consistent correlation with the AKP activity during the differentiation process, at 24 hr, 48 hr, 72 hr time points of the sodium butyrate stimulation (Figure 9E). We then performed rtRT-PCR analysis to find whether the above differentially expressed genes induced by IGFBP-rP1 in colon cancer cells (SOX9, IRS1, P15, AREG, IER5L, KRT8) were also altered in Caco2 cells during the differentiation process induced by sodium butyrate. Interestingly, consistent with the gene expression change induced by IGFBP-rP1, downregulation of IRS1 and SOX9 were also found during the differentiation process induced by butyrate. Other genes expression change patterns during the differentiation process were not in consistent with those induced by IGFBP-rP1.

## Discussion

The spatial organization of the colonic mucosa implies that in vivo the cells normally undergo a sequence of events which includes proliferation, differentiation, apoptosis, and extrusion. The continuously regenerating colonic epithelium is characterized by the proliferation of undifferentiated multipotent stem cells located at the base of the crypts and by shedding of mature fully differentiated cells at the luminal surface. It is during this translocation that differentiation occurs. Thus, to identify the differentiation associated function of IGFBP-rP1 in vivo, it was of interest to determine the localization of IGFBP-rP1-expressing cells in normal colonic epithelium. We here first reported that IGFBP-rP1 expresses specifically in the well-differentiated cells on the surface throughout the length of the intestine, strikingly matches the expression pattern of the differentiation marker of colon epithelium. Interestingly, IGFBP-rP1 expression is also well maintained and actually increased in colorectal cancer, when compared with the paired normal tissues, consistent with our previous study which performed in another collection of colon cancer samples (n = 78) [35]. The discrepancy of up regulation of IGFBP-rP1 in colon cancer tissue and low expression in cell lines may be due to the regulation of IGFBP-rP1 by the microenvironment, which is now under investigation in our laboratory. The upregulation of IGFBP-rP1 in colon cancer tissue seems paradoxical to the well defined expression of IGFBP-rP1 in terminally differentiated cells at the normal intestinal surface. Colorectal carcinoma consists of different histology types with differentiation status, including tubular adenocarcinoma, papillary carcinoma, mucinous adenocarcinoma, signet ring cell carcinoma, and undifferentiated carcinoma. The differentiation status of cancer can provide insights into the degree of malignancy, prognosis. We found that IGFBP-rP1 was strongly expressed in low grade colorectal carcinoma and weakly expressed in high grade colorectal carcinoma, indicating that the molecule is a potential differentiation-associated marker in colon cancer.

Differentiation characteristics of the colon epithelium include the emergence of a polarized morphology and the appearance of or a significant increase in brush border hydrola enzyme activities, such as AKP [36,37]. In this study, we choose the three colon cancer cell lines without endogenous IGFBP-rP1 expression, RKO, SW620 and CW2 cell lines, to observe the transfection of IGFBP-rP1 cDNA on the differentiation status of the cells. RKO and CW2 cell lines are both derived from primary colon adenocarcinoma. SW620 cell line was initiated from a lymph node metastasis from the primary colon adenocarcinoma. We found that transfection of IGFBP-rP1 cDNA could induce the cells to a more pronounced anterior-posterior polarity morphology, accompanied by the increase of AKP activity both in the cell lysates and cell supernatants.

Interestingly, analysis of other well known colon epithelium differentiation markers showed cell type specificity. CEA, a tumor-associated antigen, is widely used serum biomarker for colorectal cancer. Interestingly, it has also been shown to correlate with the differentiation state of colon. In the normal colonic mucosa, CEA expression showed crypt-surface distribution. CEA expression was strong in surface epithelial cells and goblet cells of the upper crypts, while very weak in the mid crypt and at the base[38]. Cell lines with high expression of CEA showed shuttle-shape morphologic changes with long or dendritic-like cytoplasmic processes, decreased cell growth and de novo tumor formation in nude mice xenograft. In colon cancer cell lines, it is also widely used as differentiation marker[39-43]. In our study, upregulated expression of CEA were induced by IGFBP-rP1 in SW620 and CW2 cells. No detectable expression of CEA induced by IGFBP-rP1 in RKO cells was observed. These findings suggested that the function of IGFBP-rP1 in regulating cell differentiation may be cell lineage specific. RKO cell line is a poorly differentiated colon carcinoma cell line developed by Michael Brattain. The AKP activity in RKO cells was lower than that of SW620 and CW2 cells. Although the AKP activity of PcDNA3.1(IGFBP-rP1)-RKO transfectants was higher than that of RKO cells, it was lower than that of PcDNA3.1(IGFBP-rP1)-SW620 and PcDNA3.1(IGFBP-rP1)-CW2 cells. Thus, the differentiation status may lower in RKO cells than in SW620 and CW2 cells. This may be could explain, at least in part, why the other well known differentiation marker such as CEA, could not be detected after IGFBP-rP1 transfection in RKO cells.

Interestingly, our findings were in consistent with the studies reported by Sprenger et al., which demonstrated the elongated appearance of high IGFBP-rP1 expressing clones in prostate cancer cells [14]. The authors explained that the morphological change was correlated with an increased sensitivity to undergo apoptosis. However, based on our findings which showed the cell morphological change was associated with regulations of several epithelium differentiation associated markers, we thought that the morphological change was tightly associated with the differentiation induction process.

IGFBP-rP1 is a protein with secretary character. We observed that recombinant IGFBP-rP1 stimulation could mimic the effects of the transfection of IGFBP-rP1 cDNA, although the extent may not be as high. These findings indicated that the autocrine stimulation was part of the mechanism for the secretary protein. Other mechanisms may also be responsible for the biological behaviour of IGFBP-rP1.

Normal cells differentiate to gain in the properties required for organ or tissue functions. However, the differentiation program can be distorted. Malignant cells have a differentiation block that results in an accumulation of inappropriate or abnormal cell type (i.e., anaplasia). However, this process could partly be reversed. Colon cancer cells undergo terminal differentiation in response to diverse differentiation stimuli. It has been well demonstrated that sodium butyrate, the natural product of intestinal flora, is an typical inducer of colon cancer cell line to terminally differentiated cells (reviewed in [44]). Exposure to sodium butyrate induces morphological and biochemical changes consistent with a more differentiated state. One of these cell lines, namely Caco2, has been extensive used and characterized for its ability to differentiate under sodium butyrate stimulation[45]. In our study, the upregulation of AKP activity and CEA expression confirmed the differentiation process, although the elongated polarity morphology change was not observed during the process. Different from the above RKO, SW620, and CW2 cells, Caco2 cells express endogenous IGFBP-rP1. Interestingly, upregulated expression of IGFBP-rP1 was detected during the differentiation process, which showed a good correlation with the most widely used differentiation marker AKP activity, again indicating IGFBP-rP1 a molecule associated with colon cancer differentiation

CDX2 is a key molecule for directing intestinal development and differentiation. A gradient of CDX2 expression formed in the crypt-villus axis, primarily in the villus[46]. Overexpression of CDX2 leads to growth arrest accompanied by upregulation of several markers associated with intestinal differentiation[47,48]. Decreased or absent expression of CDX2 were found in poorly differentiated colon carcinomas[49-51]. In colon cancer cell lines, it is also used as differentiation marker[52]. We analyzed the expression of CDX2 during the differentiation process. Interestingly, downregulation of CDX2 induced by butyrate treatment in Caco2 cells was found. The RKO cells showed a slight but reproducible decrease (when performed six times) in levels of CDX2. While the SW620 and CW2 cells showed no detectable regulation on the CDX2 expression after transfection of IGFBP-rP1 cDNA, suggesting that restoration of CDX2 is not required for differentiation in these cell lines. Our findings were consistent with the studies of Oualtrough et al [53]. In their study, no significant regulation of CDX2 was observed with the differentiation process in colon cell lines. No detectable gradient CDX2 expression along the axis of the crypt was found. These observations indicated that the differentiation associated function of CDX2 may act depend upon the cell type and may confer tissue specificity.

The studies presented here in vitro and in vivo demonstrated that IGFBP-rP1 was a potential molecule associated with colon epithelium cells differentiation, expanded the previous findings on the molecule's proliferation inhibition, apoptosis and senescence induction role. Based on different assumptions, several opposite models linking proliferation, cell death, and differentiation are currently coexisting [54]. Cellular proliferation, differentiation, apoptosis and senescence are physiological processes that show overlapping properties [55-57]. Furthermore, several lines of evidence suggested that they are alternative, independent phenomena [58,59]. The balance among proliferation, differentiation, senescence and apoptosis tightly regulated to maintain homeostasis of colon epithelium. Our findings extend our knowledge on IGFBP-rP1's role in this balance.

In this context, it is important to precisely identify the molecular signature for IGFBP-rP1 in colon cancer. Among the 78 reproducible differentially expressed genes identified, there were several genes whose altered expression induced by IGFBP-rP1 has been previously reported in prostate cancer cells by sprenger et al [32], such as IL8, KRT8. However, interestingly, SOX9 was found to be upregulated in IGFBP-rP1-transfected prostate cancer cells, while downregulated in IGFBP-rP1 transfected colon cancer cells, indicating the cell lineage specific regulation. DAG of the enriched GO categories demonstrated that the enzyme regulator activity genes together with cytoskeleton and actin binding genes were of great significance. The enzyme activity participated in many biomedical processes. The relation between the cytoskeleton and the differentiation process has been well demonstrated in different organ systems[60]. Our findings provide a clue for IGFBP-rP1's possible function in these important biomedical processes. The upreguation of TAGLN and downregulation of SOX9, IRS1, P15, AREG, IER5L, KRT8 in RKO, SW620 and CW2 colon cancer cells indicated these genes may be the target molecules for the biological behaviour of IGFBP-rP1 in colon cancer. The sodium butyrate induced Caco2 differentiation process was accompanied by downregulation of IRS1 and SOX9. While other IGFBP-rP1 responsible genes exhibited different expression patterns via induction by sodium butyrate. It has been demonstrated that sodium butyrate induce cellular growth arrest, differentiation and apoptosis in colon cancer cells through various molecular mechanisms, including histone hyperacetylating [61,62], nuclear factor kappaB (NF-kB) activation together with a defective beta1 integrin- focal adhesion kinase (FAK)- phosphatidylinositol 3'-kinase (PI3K) pathways signaling[63], downregulating extracellular signal-regulated kinase (ERK) phosphorylation [64] and induction of cyclin D3 and p21 expression[65]. Our findings indicated that IGFBP-rP1 possiblely worked in different signaling pathways during the differentiation process. Our observations are consistent with the studies by Velcich et al. demonstrating that different inducers (12-O-tetradecanoylphorbol-13-acetate, forskolin, and sodium butyrate) modulate specific sets of markers in the process of differentiation induction, suggesting various inducers seem to utilize different intracellular pathways for induction of differentiation [39].

Downregulation of IRS1 and SOX9 by both IGFBP-rP1 and sodium butyrate in colon cancer cells indicated that these two genes may play important roles in the differentiation and apoptosis induction process in colon epithelium. IRS1 was a docking protein for both type 1 insulin-like growth factor receptor (IGF-IR) and insulin receptor. It is a key mediator of the actions of insulin and IGFs, sending a mitogenic, anti-apoptotic, and anti-differentiation signal [66,67]. IRS-1 null mice exhibit reduced body growth and reduced growth of several organs including the intestine [68]. Up regulation of IRS1 were found in colon cancer, while downregulation of IRS1 levels were found in differentiating cells [69,70]. As to SOX9, previous studies showed the role of SOX9 in committed differentiation, such as chondrocyte differentiation, outer root sheath differentiation, and the formation of the hair stem cell compartment. Blache and colleagues reported that SOX9 can inhibit intestinal crypt differentiation in the colon [71]. Contrary to the expression pattern of IGFBP-rP1 in the healthy human colon epithelium, our research group found that the expression of SOX9 is restricted to the proliferative, lower half of the crypt [72], consistent with the studies by Bastide et al.[73]. Additional experiments demonstrating the exact roles of these two genes in mediating IGFBP-rP1's effect on differentiation should be performed.

Analysis of differentiation by tumor cells often provides valuable information for both the diagnosis and therapy of human cancers. One can envision very exciting times in the future as seeks to testing the role of IGFBP-rP1 using the knockout mice model. Because our current results are limited to colorectal tumorigenesis models, other studies will be needed to determine whether our findings apply to other organ systems. From a clinical point of view, specifically targeting and manipulating the function of IGFBP-rP1 may offer a novel approach to the differentiation therapy of colon cancer. Further experiments are under way to study the molecular mechanism underlying the observations reported here.

## Conflict of interests

The authors declare that they have no competing interests.

## Authors' contributions

RWJ carried out the design of the study, the microarray analysis and the realtime PCR analysis, participated in the cell morphology observation assay, differentiation marker analysis assay, ELISA assay, immunohistochemistry result analysis assay and the statistical analysis, drafted the manuscript. ZSZ participated in the expression analysis of well known differentiation markers, cell morphology change observation, realtime PCR assay. WHB participated in the cell differentiation assay. XFY collected the tissue samples and carried out the immunohistochemistry staining. DH participated in the analysis of the immunohistochemistry result. MY participated in the cell culture and western blot analysis. LMD participated in the design of the study and helped drafting the manuscript. All authors read and approved the final manuscript.
